# Preparation of Ternary Solid Waste-Based Composite Cementitious Material and Its Performance in Stabilized Gravel

**DOI:** 10.3390/ma19132870

**Published:** 2026-07-05

**Authors:** Yifei Wang, Lihua Zhong, Jian Sun, Haojie Ji, Wei Chen, Zunqing Liu

**Affiliations:** 1 School of Traffic and Logistics Engineering, Xinjiang Agricultural University, Urumqi 830052, China; wangyifei808@163.com (Y.W.); jihaojie@xjau.edu.cn (H.J.); 2 Key Laboratory of Transportation and Logistics Engineering in Xinjiang, Xinjiang Agricultural University, Urumqi 830052, China; 3 Xinjiang Communications Construction Group Co., Ltd., Urumqi 830016, China

**Keywords:** fly ash, ground granulated blast-furnace slag, carbide slag, semirigid base course, microstructure

## Abstract

To support the achievement of the carbon peaking and carbon neutrality goals and promote the resource utilization of industrial solid waste, a ternary solid waste composite cementitious material was prepared by blending ground granulated blast-furnace slag (GGBFS), fly ash (FA), and carbide slag (CS) with cement. The optimal mix ratio was determined through single-factor experiments and response surface methodology. The synergistic hydration mechanism was elucidated using microstructural characterization techniques, including XRD, FTIR, TG-DTG, and SEM. The composite material was then applied to a semirigid base course, and its mechanical properties and durability were systematically evaluated. The results indicate that the optimal levels of FA, GGBFS, and CS investigated in the single-factor experiments are 20–40%, 30–50%, and 2–6%, respectively. The optimal mix ratio of the ternary solid waste composite is 21.0% FA, 36.3% GGBFS, and 5.7% CS. The underlying microstructural mechanism is that carbide slag creates a highly alkaline environment, which activates the pozzolanic activity of GGBFS and fly ash, leading to the formation of hydration products dominated by C-(A)-S-H gel. With increasing curing age, the gel structure evolves from a loose and disordered state to a dense and ordered state, ultimately forming a compact microstructure based on a highly polymerized C-(A)-S-H gel matrix. The 7-day unconfined compressive strength of the stabilized gravel using the solid waste-based composite cementitious material reached 5.93 MPa, and the 28-day drying shrinkage coefficient was reduced by 18.3% compared with that of cement-stabilized gravel. After 18 freeze–thaw cycles, the compressive strength increased by 2.4%, with the pore structure characterized by a “macropores decreasing, micropores increasing” refinement pattern. After 18 wetting–drying cycles, the cumulative strength loss was 11.26%, outperforming cement-stabilized gravel. Combined with SEM observations, these performance improvements are attributed to the densely intertwined hydration products, particularly C-S-H gel, which effectively fill the voids between aggregate particles and significantly enhance the volume stability, freeze–thaw resistance, and wetting–drying durability of the stabilized gravel. The application of this cementitious material in a semirigid base course demonstrates excellent mechanical and durability properties, providing a theoretical basis and technical support for the widespread application of industrial solid waste in road engineering.

## 1. Introduction

With the acceleration of industrialization, the generation of industrial solid waste, such as steel slag, fly ash, and ground granulated blast-furnace slag, has increased substantially [1,2,3]. As a major industrial nation, China has accumulated approximately 60 billion tons of solid waste over the years, with an annual generation of nearly 3.6 billion tons, and the growth rate remains high [4]. The accumulation of vast quantities of solid waste not only represents a massive waste of potential resources but also occupies extensive tracts of land and imposes a severe environmental burden [5]. Furthermore, carbon emissions from the cement industry account for approximately 25% of global industrial CO_2_ emissions [6], creating an increasingly acute contradiction with the carbon peaking and carbon neutrality goals. Against this backdrop, the synergistic coupling of multi-component solid waste to produce low-carbon cementitious materials as a substitute for cement has emerged as a strategic pathway for the green and low-carbon transformation of the construction industry, as it simultaneously enables the utilization of industrial solid waste and reduces carbon emissions in building material production. In recent years, research on the component design, activation methods, and hydration mechanisms of multi-solid-waste cementitious materials has continued to deepen and their application in concrete and road engineering increasingly explored, attracting significant attention from both the international academic and industrial communities.

In research on binary solid waste cementitious materials, synergistic combinations can be designed based on the chemical composition and mineralogical characteristics of individual solid wastes [7,8]. For instance, ground granulated blast-furnace slag is rich in active components such as CaO, SiO_2_, and Al_2_O_3_, which can rapidly undergo hydration reactions in an alkaline environment to form cementitious products [9]. The cementitious properties of fly ash primarily stem from the pozzolanic reactions of its active SiO_2_ and Al_2_O_3_ under alkaline conditions, generating gels such as C-S-H and C-A-H [10]. Carbide slag typically contains over 65% CaO and exhibits strong alkalinity upon contact with water, making it suitable for use as an alkali activator [11,12,13,14]. When blended with pozzolanic materials, the system’s strength primarily derives from the hydraulic cementation and filling effects between carbide slag and the pozzolanic components [15]. Owing to this characteristic, carbide slag is often blended with pozzolanic materials such as ground granulated blast-furnace slag and fly ash to activate their reactivity. In research on binary solid waste cementitious systems, Li et al. [16] utilized carbide slag to activate blast-furnace slag and found that carbide slag accelerates the hydration process of ground granulated blast-furnace slag, promotes the dissolution of the glassy matrix, and generates various hydration products such as C-S-H, C-A-H, C-A-S-H, AFt, AFm, and hydrotalcite. However, excessive carbide slag content inhibits the formation of hydration products, leading to an increase in Ca(OH)_2_ crystal content and a consequent decrease in strength. Vichan et al. [17] used a blend of biomass ash and carbide slag to solidify soft clay and found that the synergistic interaction between the two materials stimulates the pozzolanic reaction, generating products such as ettringite and calcium aluminate hydrate, which results in a denser microstructure of the solidified soil and a significant increase in unconfined compressive strength.

In research on ternary and multi-component solid waste synergistic systems, Yang et al. [18] constructed a magnesium–coal ash-based cementitious system using modified magnesium slag, coal gasification slag, and desulfurization gypsum. They found that the ternary synergistic effect was relatively weak in the early stages, but in the later stages, the depolymerization of silicoaluminates in coal gasification slag, the leaching of sulfates from desulfurization gypsum, and the interaction with the hydration products of modified magnesium slag promoted the formation of C-(A)-S-H gels and ettringite, thereby enhancing compressive strength. Zhang et al. [19] confirmed the existence of significant synergistic effects among steel slag, red mud, and ground granulated blast-furnace slag through analyses of strength, phase composition, thermogravimetric behavior, microstructure, and pore structure. The improvement in mechanical properties was attributed to the formation of C-S-H gel and ettringite, as well as the densification of the pore structure. Shen et al. [20] revealed the synergistic coupling mechanisms among lithium slag, fly ash, silica fume, and slag from three perspectives: the evolution of precursor reactivity, gel microstructure, and macroscopic properties. Wang et al. [21] investigated the effects of five factors, including the steel slag-to-slag ratio and the dosage of desulfurization gypsum on the performance of alkali-activated ternary solid waste cementitious materials, finding that desulfurization gypsum is a key factor in enhancing early-age strength. Ge et al. [22] prepared multi-component solid waste cementitious materials using steel slag, ground granulated blast-furnace slag, desulfurization gypsum, fly ash, and cement as raw materials. Through orthogonal experiments to optimal mix proportions, the 28-day compressive strength was increased by 7.6% compared to the pure cement group, while drying shrinkage and chloride ion permeability resistance were improved by 22.9% and 9.8%, respectively. Microscopic analysis indicated that the performance improvements stemmed from the synergistic effects of the solid waste materials, which promoted the formation of ettringite and C-(A)-S-H gels.

Considerable efforts have been devoted to the synergistic effects and mix optimization of multi-component solid waste-based cementitious materials, with notable progress in ternary and higher-order systems. However, most existing ternary systems are centered on sulfate-based activation or the blending of multiple industrial by-products, where the synergistic mechanisms primarily rely on sulfate–aluminate reactions to form ettringite or on complementary hydration among different waste components. In contrast, the ternary system composed of carbide slag, fly ash, and ground granulated blast-furnace slag is fundamentally distinct in its activation mechanism: carbide slag creates a highly alkaline environment that depolymerizes the glassy structure of ground granulated blast-furnace slag and triggers the pozzolanic reaction of fly ash, with the coupling effect governed by the synergy between alkali activation and pozzolanic reactivity. Most existing studies have focused primarily on hydration mechanisms and mechanical properties of the cementitious materials themselves, with limited attention to their systematic application in semirigid base courses, particularly regarding comprehensive evaluations of both mechanical performance and durability. In this context, this study utilized carbide slag, fly ash, and ground granulated blast-furnace slag as the primary raw materials combined with ordinary Portland cement to formulate a ternary solid waste-based composite cementitious material. The mix proportions were optimized through single-factor tests and response surface methodology to identify the effect of each component on compressive strength and determine the optimal dosage ranges. The hydration product evolution and synergistic coupling mechanism under carbide slag activation were elucidated using X-ray diffraction, Fourier-transform infrared spectroscopy, thermogravimetric–derivative thermogravimetric analysis, and scanning electron microscopy. The optimized material was then applied to semirigid base courses, where its mechanical properties, drying shrinkage, and thermal shrinkage characteristics, as well as freeze–thaw and wetting–drying durability, were systematically evaluated. This study thus extends the research on carbide slag-based ternary cementitious materials from material-level mechanistic analysis to comprehensive performance assessment in semirigid base applications, establishes a systematic linkage between the alkali activation–pozzolanic synergy and base-layer engineering performance, and provides both theoretical foundation and technical guidance for the efficient utilization of carbide slag-bearing solid wastes in road infrastructure.

## 2. Materials and Methods

### 2.1. Raw Materials

**Figure 1:**
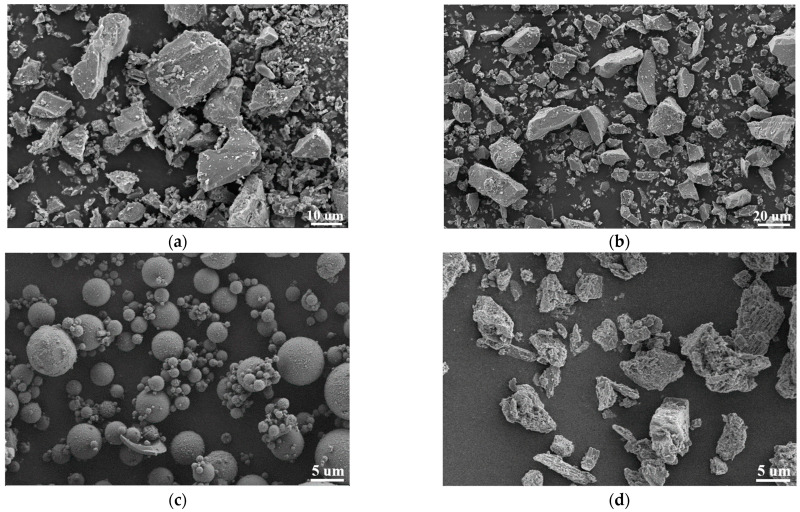
SEM micrographs of raw materials. (**a**) SEM micrograph of cement; (**b**) SEM micrograph of GGBFS; (**c**) SEM micrograph of FA; (**d**) SEM micrograph of CS.

The cement used in this test was P·O 42.5 ordinary Portland cement manufactured by Xinjiang Tianshan Materials Co., Ltd., Urumqi, China. The chemical composition of this cement, determined by X-ray fluorescence (XRF) spectrometry using the fused glass bead method, is shown in Table 1, the physical properties are shown in Table 2, and an SEM micrograph is shown in Figure 1a.

**Figure 2:**
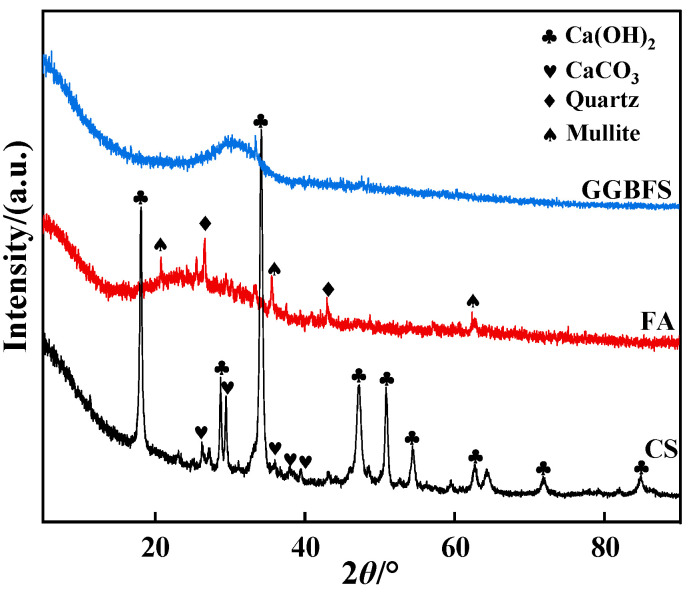
XRD of solid waste materials.

The ground granulated blast-furnace slag (GGBFS, S95 grade) used in this study was supplied by Xinjiang Bayi Iron and Steel Co., Ltd., Urumqi, China. An SEM micrograph is shown in Figure 1b. The GGBFS particles are predominantly irregular in shape, with glassy blocky and flaky morphologies and a wide particle size distribution. The XRD pattern in Figure 2 exhibits no distinct diffraction peaks, confirming that the GGBFS is mainly composed of amorphous glassy phases. The fly ash (FA) was Class F Grade II, obtained from Xinjiang Changji Junheng Building Materials Co., Ltd., Changji, China. The SEM image in Figure 1c reveals that the FA particles are mainly smooth spheres or near-spheres of relatively uniform size, along with minor flocculent aggregates. The XRD pattern in Figure 2 shows a broad amorphous hump superimposed with sharp crystalline peaks assigned to quartz and mullite, indicating the coexistence of amorphous and crystalline phases in the FA. The carbide slag (CS) was sourced from Xinjiang Zhongtai Chemical Co., Ltd., Urumqi, China. Before testing, the CS was ground and sieved. Its SEM morphology and XRD pattern are shown in Figure 1d and Figure 2, respectively. The chemical compositions of GGBFS, FA, and CS are summarized in Table 3, all determined by X-ray fluorescence (XRF) spectrometry using the fused glass bead method.

The standard sand used in this study was produced by Xiamen ISO Standard Sand Co., Ltd., Xiamen, China. The aggregate selected was graded gravel sourced from the Xinjiang Changji Zhundong aggregate yard. The performance indicators of the aggregate are presented in Table 4.

### 2.2. Methods

#### 2.2.1. Mix Design of Solid Waste Composite Cementitious Materials

The mix design and strength testing followed the Chinese standard GB/T 17671-2021 (ISO method) [23]. Mortar mixtures were prepared with a water-to-binder ratio of 0.5 and a binder-to-sand ratio of 1:3, cast into 40 mm × 40 mm × 160 mm prisms using a triple-gang mold. FA, GGBFS, and CS (added as an external alkali activator) contents were chosen as the variables. For each mix, three prisms were cast for flexural strength and six halves for compressive strength, as specified by the standard. All reported strength values are the average of the replicate measurements. The experimental program consisted of the following.

(1) Single-factor experiments: The compressive strength and flexural strength of mortar specimens at 3-day, 7-day, and 28-day curing ages were determined for each mix proportion to preliminarily establish the suitable incorporation ranges of the three solid waste materials. The experimental mix proportions are shown in Table 5.

(2) Response surface optimization: Based on the single-factor experimental results, a response surface model was established using Design-Expert 13 software. The contents of FA, GGBFS, and CS were taken as independent variables, with the specific factor levels shown in Table 6. The 28-day compressive strength, 28-day flexural strength, and fluidity were selected as the response values to optimize the mix proportions and determine the optimal combination.

#### 2.2.2. Performance of Gravel Base Course Stabilized with Solid Waste Composite Cementitious Materials

**Figure 3:**
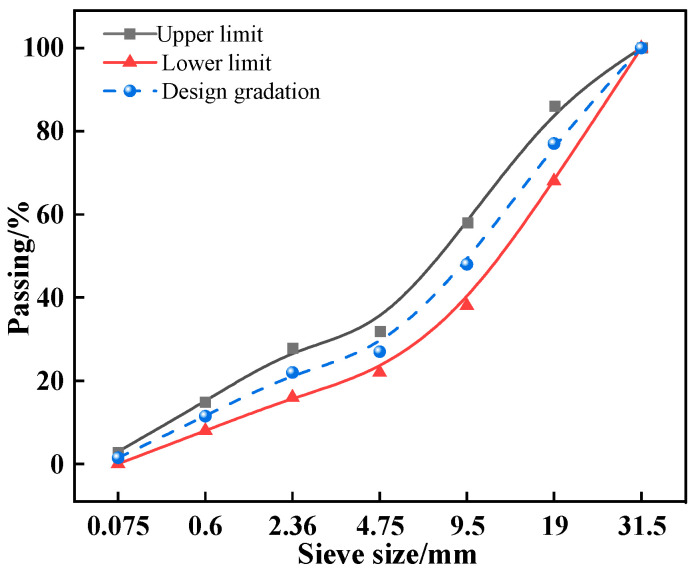
Grading curve.

(1) In accordance with the Technical Guidelines for Construction of Highway Roadbases (JTG/T F20-2015) [24] and the Test Methods of Materials Stabilized with Inorganic Binders for Highway Engineering (JTG 3441-2024) [25], the C-B-3 gradation type was adopted, and the gradation curve is shown in Figure 3. To determine the optimal binder content, four dosage levels—3%, 4%, 5%, and 6%—were designed. The maximum dry density and optimum moisture content at each dosage level were determined through standard Proctor compaction tests, and the results are presented in Table 7. Cylindrical specimens of ϕ 150 mm × 150 mm were prepared by static compaction using the obtained compaction parameters. Both unconfined compressive strength and splitting tensile strength tests were conducted in accordance with JTG 3441-2024, with nine replicates prepared for each mechanical test and the reported values representing the average of the replicate measurements.

(2) Based on the mechanical property test results, the optimal binder content was determined to be 5%. The experimental group consisted of gravel stabilized with 5% solid waste composite cementitious material (hereinafter referred to as SWCCM-stabilized gravel, SWCCM-SG), while the control group was prepared using gravel stabilized with 4% ordinary Portland cement (P·O 42.5) (cement-stabilized gravel, CSG). The cement content of 4% in the control group was selected within the recommended dosage range for cement-stabilized base courses specified in JTG/T F20-2015, and is considered representative of engineering practice. The specimens from both groups were subjected to the following durability tests.

Volumetric stability tests: In accordance with JTG 3441-2024, medium beam specimens of 100 mm × 100 mm × 400 mm were prepared using the static compaction method. After 7 days of standard curing, the drying shrinkage test and thermal shrinkage test were conducted following the T0854-2024 and T0855-2024 methods, respectively.

Freeze–thaw cycle test: Cylindrical specimens of ϕ 150 mm × 150 mm were prepared and tested in accordance with the T0858-2009 method. The freeze–thaw regime consisted of freezing at −18 °C for 16 h and thawing in a 20 °C water bath for 8 h. The height and mass of each specimen were measured before and after each freeze–thaw cycle.

Wet–dry cycle test: The test was conducted in accordance with the T0582-2020 method specified in the Test Methods of Cement and Concrete for Highway Engineering (JTG 3420-2020) [26]. Specimens cured under standard conditions for 26 days were oven-dried at 65 °C ± 5 °C for 48 h, cooled, then immersed in water for 15 h. After removal, they were air-dried naturally for 1 h, then oven-dried at 80 °C ± 5 °C for 6 h, and finally cooled to room temperature to complete one cycle.

#### 2.2.3. Microstructural Characterization of Solid Waste Composite Cementitious Materials

(1) X-ray diffraction (XRD): XRD analysis was carried out on a Rigaku Smart Lab SE diffractometer (Rigaku Corporation, Akishima, Tokyo, Japan) using Co Kα radiation to identify the phase composition and crystalline structure of the samples. For sample preparation, the dried materials were ground to pass through a 75 μm sieve and then pressed flat into the specimen holder. Diffractograms were recorded over a 2θ range of 5° to 90° at a scan rate of 2°/min. Phase identification and peak assignment were performed with Jade 9 software. The evolution of hydration products and crystalline phases was examined by comparing the diffraction patterns of samples with different curing ages and mix proportions.

(2) Fourier-transform infrared spectroscopy (FTIR): FTIR spectra were collected on a Thermo Fisher Scientific Nicolet iS20 spectrometer (Thermo Fisher Scientific, Waltham, MA, USA) to characterize the chemical bonds and functional groups in the samples. To prepare the specimens, a small amount of dried sample was mixed with KBr powder at an appropriate ratio, ground thoroughly, and pressed into transparent pellets. The spectra were recorded over a wavenumber range of 4000 to 400 cm^−1^ with a resolution of 4 cm^−1^. The position and intensity of characteristic absorption bands, corresponding to Si–O, Al–O, and C–O vibrations, were used to identify the reaction products and to assess their degree of polymerization.

(3) Thermogravimetric (TG) analysis: TG measurements were performed on a TA Instruments TGA 550 (TA Instruments, New Castle, DE, USA) under a nitrogen atmosphere with a flow rate of 25 mL/min. The dried powder samples were ground to a homogeneous state, and approximately 5–10 mg of each was placed in an alumina crucible prior to testing. The samples were heated from room temperature to 800 °C at a rate of 10 °C/min, and the mass loss was continuously recorded as a function of temperature. The contents of free water, bound water, Ca(OH)_2_, and CaCO_3_ in the samples were quantified based on the mass losses observed over different temperature intervals.

(4) Scanning electron microscopy (SEM): Microstructural observations were conducted on a Zeiss Sigma 300 field-emission scanning electron microscope (Carl Zeiss AG, Oberkochen, Germany). The dried samples were mounted on conductive tape. Owing to their poor conductivity, the specimens were sputter-coated with gold before imaging. Secondary electron images were acquired at an accelerating voltage of 5 kV and a working distance of approximately 8–10 mm at various magnifications to examine the morphology, distribution, and densification of the hydration products.

(5) Mercury intrusion porosimetry (MIP): Pore structure characterization was performed using a Micromeritics AutoPore V 9600 automatic mercury porosimeter (Micromeritics Instrument Corporation, Norcross, GA, USA). For sample preparation, the hardened paste was crushed into particles of approximately 5 mm in size, dried, and then sealed in a penetrometer for analysis. The pressure ranged from 0.23 to 33,000.50 psi, corresponding to pore diameters from 774.29 μm to 5.48 nm. The cumulative mercury intrusion, pore size distribution, and total porosity were analyzed to evaluate the pore structure characteristics and the microstructural compactness of the materials.

## 3. Mechanical and Microstructural Characterization of Solid Waste Cementitious Materials

### 3.1. Results of Single-Factor Experiments

**Figure 4:**
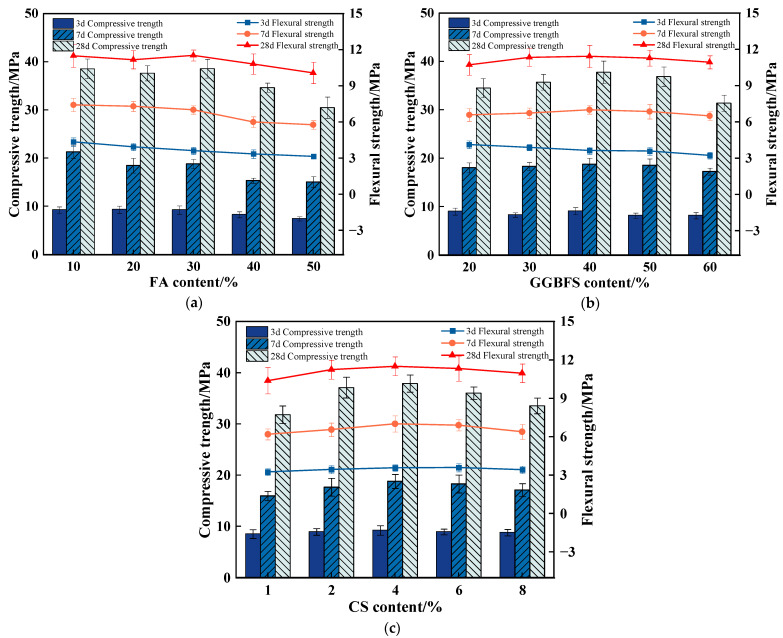
Single-factor experimental results. (**a**) Effect of FA content on the mechanical properties of solid waste composite cementitious mortar. (**b**) Effect of GGBFS content on the mechanical properties of solid waste composite cementitious mortar. (**c**) Effect of CS content on the mechanical properties of solid waste composite cementitious mortar.

Figure 4 presents the mechanical properties of the solid waste composite cementitious mortar specimens with varying solid waste contents and curing ages. Overall, as the contents of FA, GGBFS, and CS increased, both the compressive and flexural strengths initially increased and then decreased. In addition, the strength increased significantly with prolonged curing age.

As shown in Figure 4a, when the FA content was 30%, the 28-day compressive and flexural strengths reached their maximum values of 38.58 MPa and 11.52 MPa, respectively. At 3 days, the strength remained relatively stable within the FA content range of 10–30%, but decreased markedly when the content increased to 40% and 50%. At 28 days, the strength within the 10–30% range varied between 37.56 MPa and 38.58 MPa, suggesting that the pozzolanic effect of FA effectively compensated for the strength loss caused by the reduction in cement clinker. However, when the FA content reached 40%, the strength decreased by 8.38%, with further deterioration at 50%. Based on these results, the optimal FA content range was determined to be 20–40%.

Figure 4b shows that the optimum mechanical performance of GGBFS occurred within the content range of 30–40%, with the 28-day compressive strength peaking at 40% GGBFS content. The 3-day strength showed no clear trend with increasing GGBFS content, fluctuating within a narrow range, reflecting a dynamic balance between the early-age hydration activity and the cement dilution effect. At 7 days, the strength remained relatively stable within the 20–50% range, but decreased significantly at 60%. At 28 days, the strength increased continuously as the content increased from 20% to 40%, whereas at 60% content, it decreased by 17% compared with that at 40%. The flexural strength also reached its maximum at 40% GGBFS content. These results indicate that an appropriate amount of GGBFS generates a synergistic hydration effect with FA: insufficient GGBFS limits the availability of reactive phases, while excessive GGBFS reduces the hydration driving force. Therefore, the optimal GGBFS content range was determined to be 30–50%.

From Figure 4c, the strength at each curing age increased continuously as the CS content increased from 1% to 4%, with the 28-day compressive strength rising from 31.75 MPa to a peak of 37.83 MPa. When the CS content increased to 6%, the strength decreased slightly, but remained at a relatively high level. At 8% CS content, the strength deteriorated markedly, with the 28-day compressive strength decreasing by 11.4% compared with that at 6%. These findings suggest that CS, as an alkali activator, effectively stimulates the pozzolanic activity of GGBFS and FA at an appropriate dosage, promoting additional C-S-H gel formation and leading to a denser microstructure. Insufficient CS content provides inadequate alkalinity and thus a limited activation effect, whereas excessive CS content results in overly high alkalinity, causing rapid, but loose formation of hydration products. This may induce adverse effects such as efflorescence or delayed ettringite formation (DEF), compromising long-term strength. Based on the comprehensive mechanical properties at all curing ages, the optimal CS content range was determined to be 2–6%.

### 3.2. Response Surface Optimization of Mix Proportions

**Figure 5:**
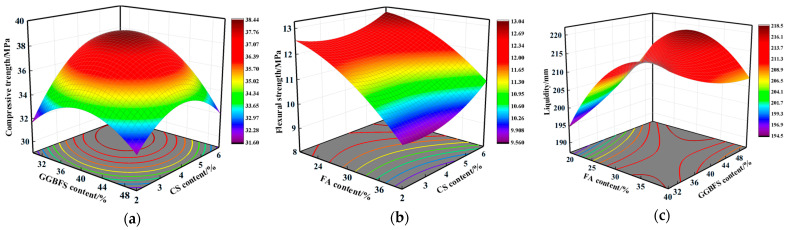
Response surface plots of interactive effects. (**a**) Interactive effect of GGBFS content and CS content on 28-day compressive strength. (**b**) Interactive effect of FA content and CS content on 28-day flexural strength. (**c**) Interactive effect of FA content and GGBFS content on fluidity.

Based on the single-factor experiments described in Section 3.1, relatively suitable content ranges for each factor were obtained. In accordance with the Box–Behnken experimental design of response surface methodology, the contents of FA, GGBFS, and CS were selected as the response factors, denoted X_1_, X_2_, and X_3_, respectively. The response variables were the 28-day compressive strength (Y_1_), 28-day flexural strength (Y_2_), and fluidity (Y_3_) of the mortar specimens prepared with the solid waste composite cementitious material. A total of 17 experimental runs were conducted. Based on the experimental results, quadratic regression models for Y_1_, Y_2_, and Y_3_ were established, as shown in Equations (1)–(3). The analysis of variance (ANOVA) results for the models are presented in Table 8. As shown, the models were overall highly significant (*p* < 0.0001) with no significant lack of fit (*p* > 0.05). The adjusted R^2^ and predicted R^2^ values were in close agreement, indicating no overfitting and strong predictive capability. Thus, the models are statistically reliable for mix optimization and performance prediction. The response surface plots illustrating the interactive effects of the factors on the response values are presented in Figure 5.

(1) $$\begin{array}{c} Y_{1}=37.83-3.03X_{1}-1.31X_{2}+1.49X_{3}+0.085X_{1}X_{2}-0.4725X_{1}X_{3}-1.36X_{2}X_{3}\\ +2.23X_{1}^{2}-2.29X_{2}^{2}-2.38X_{3}^{2} \end{array}$$

(2) $$\begin{array}{c} Y_{2}=11.48-1.29X_{1}-0.8925X_{2}+0.4138X_{3}+0.1975X_{1}X_{2}+0.165X_{1}X_{3}\\ +0.3175X_{2}X_{3}-0.447X_{1}^{2}-0.8445X_{2}^{2}+0.458X_{3}^{2} \end{array}$$

(3) $$\begin{array}{c} Y_{3}=213.84+4.09X_{1}+2.35X_{2}-12.89X_{3}-6.58X_{1}X_{2}+10.1X_{1}X_{3}+2.63X_{2}X_{3}\\ -8.37X_{1}^{2}+2.05X_{2}^{2}+4.13X_{3}^{2} \end{array}$$

As shown in Figure 5a, when the FA content was held at the central level, the 28-day compressive strength first increased and then decreased as the GGBFS or CS content increased. A comparison of the two curves reveals that the CS curve is steeper, and the contour lines are denser along the CS direction, indicating that CS content has a more significant influence on compressive strength. The compressive strength reached a peak value of 37.59 MPa at a GGBFS content of 40% and a CS content of 4%. As shown in Figure 5b, the flexural strength tended to decrease with increasing GGBFS content, whereas it increased slightly at first and then more sharply with increasing CS content. When GGBFS was held at the central level, increasing CS from 2% to 6% raised the flexural strength by 14.6%. Conversely, when CS was held at the central level, increasing GGBFS from 30% to 50% reduced the flexural strength by 20.0%. The contour lines were denser along the GGBFS direction, indicating that GGBFS content exerted a more pronounced effect on flexural strength. As shown in Figure 5c, the three-dimensional response surface for fluidity exhibited a distinct convex morphology with a peak region. The fluidity reached a maximum value of approximately 215.5 mm when the FA content was at a high level and the GGBFS content was at a low level, corresponding to the optimal workability of the mortar. The contour lines displayed a pronounced elliptical profile, suggesting a strong interaction between FA and GGBFS. At a fixed GGBFS content, the fluidity increased rapidly at first and then decreased gradually with increasing FA content. At a fixed FA content, the fluidity first decreased and then increased with increasing GGBFS content. This reflects a dual-influence mechanism: an appropriate combination of GGBFS and FA can optimize particle gradation and release free water through a composite filling effect, whereas an excessive amount of either component disrupts the packing density or increases the water demand, leading to a decline in fluidity.

The Numerical module in Design-Expert 13 software was used for multi-objective optimization to determine the optimal FA, GGBFS, and CS contents for maximizing the overall performance of the solid waste composite cementitious mortar. The optimized mix proportions were FA 21.0%, GGBFS 36.3%, and CS 5.7%. Under this mix proportion, the 28-day compressive strength was 42.93 MPa, the 28-day flexural strength was 13.02 MPa, and the fluidity was 184.1 mm. The model predictions were validated through five replicate experiments, with relative errors between the measured and predicted values for all response variables within 5%. The optimized CS content is generally consistent with the optimal dosage ranges reported for comparable ternary systems in the literature [27,28], confirming the reliability of the optimization. Notably, despite a considerably higher total content of FA and GGBFS (approximately 57%) than in some reported studies, the 28-day compressive strength of the developed system reaches 42.93 MPa, surpassing that of similar ternary solid waste-based systems [21,22]. These comparisons further demonstrate that response surface methodology can effectively identify mix proportions that combine a reasonable CS dosage with favorable mechanical properties, validating its applicability for multi-objective optimization of ternary solid waste-based cementitious materials.

### 3.3. Microstructural Mechanism Analysis of Solid Waste Composite Cementitious Materials

To elucidate the hydration product evolution and microstructural densification mechanism of the solid waste composite cementitious material under the optimal mix proportion, paste specimens were prepared using the optimal mix proportion obtained from response surface optimization and cured for 7 days and 28 days, respectively. The phase composition, functional group structure, thermal stability, and microstructure of the hydration products were systematically characterized using XRD, FTIR, TG-DTG, and SEM to explore the intrinsic relationship between the microstructural characteristics and the macroscopic mechanical properties.

#### 3.3.1. Phase Composition of Hydration Products

**Figure 6:**
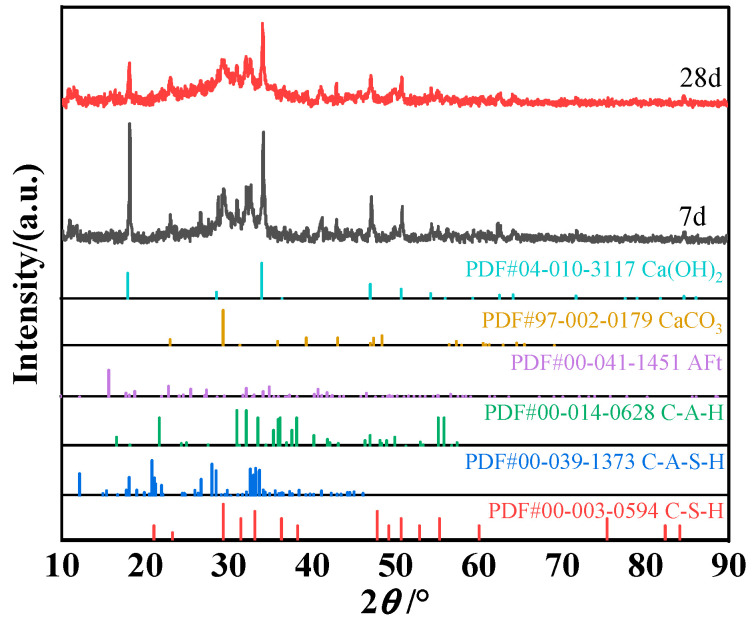
XRD pattern of the solid waste composite cementitious material.

Figure 6 presents the XRD patterns of the solid waste composite cementitious material at curing ages of 7 days and 28 days. The most prominent diffraction features appear at approximately 2θ = 18.0° and 34.8°, corresponding to the main diffraction peaks of calcium hydroxide (CH), indicating an extremely high crystallinity of CH in the system. The abundant presence of CH is primarily attributed to the incorporation of carbide slag, which not only fills pores as a physical micro-filler but also serves as an alkaline activator, providing a sustained and highly alkaline calcium-rich chemical environment for the depolymerization of the aluminosilicate glass network in GGBFS and FA [29]. A comparison of the 7-day and 28-day patterns reveals that the absolute intensities of the main CH diffraction peaks decreased to a certain extent at 28 days. This change is attributed to the progression of hydration: on the one hand, the dissolution rates of active SiO_2_ and Al_2_O_3_ from GGBFS and fly ash accelerate, leading to a gradual increase in the consumption of CH by the pozzolanic reaction; on the other hand, the abundant C-(A)-S-H gel formed physically encapsulates the CH crystal surfaces, shielding them from X-ray diffraction to some extent. The combined effect of these two processes manifests as a relative reduction in the CH peak intensities. In the 2θ range of 25–35°, the 7-day pattern exhibits a diffuse hump [30], which originates from the strong diffuse scattering of the low-polymerization, highly disordered amorphous C-(A)-S-H gel generated during the early stage when hydration is still insufficient. At 28 days, the diffuse envelope tends to broaden, indicating that as hydration proceeds, the C-(A)-S-H gel structure becomes denser and more ordered and its diffuse scattering effect weakens [31]. The decrease in the CH crystalline peak intensity and the broadening of the gel diffuse hump are temporally synchronous and unified within the same hydration process: the continuous dissolution of CH provides the necessary calcium source and alkalinity for the enhancement of gel polymerization, while the increased ordering of the gel in turn accelerates the consumption of CH in the interfacial transition zone by the pozzolanic reaction. These two processes synergistically drive the evolution of the system toward a dense network skeleton.

#### 3.3.2. Characteristics of Chemical Bonds and Functional Groups

**Figure 7:**
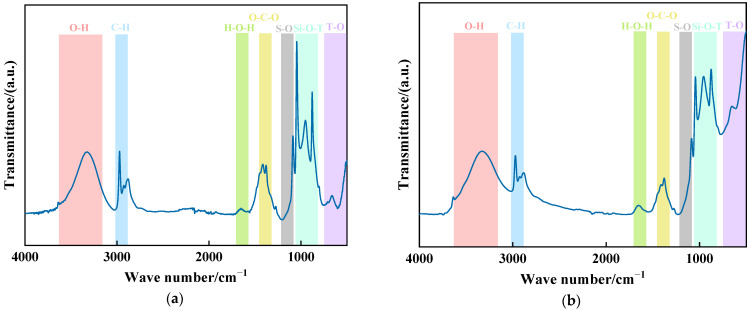
FTIR spectra of the solid waste composite cementitious material: (**a**) 7 days; (**b**) 28 days.

Figure 7 presents the FTIR spectra of the solid waste composite cementitious material at curing ages of 7 days and 28 days. The presence of the free –OH stretching vibration band near 3440 cm^−1^ [32,33] corroborates the CH crystalline peaks observed in the XRD patterns, confirming the persistent presence of CH in the system. The O–C–O vibration band at 1450 cm^−1^ and the composite characteristic band near 1100 cm^−1^, dominated by Si–O–T (T = Si or Al) vibrations with superimposed S–O absorption, remain stable, reflecting the development of the C-(A)-S-H gel framework and the coexistence of minor carbonate and sulfate species [34,35]. A comparison of the 7-day and 28-day spectra reveals a distinct evolution of the vibration band profile in the range of 900–1200 cm^−1^. At 7 days, this region displays a pattern characterized by “one dominant peak with two subordinate peaks,” indicating that the early-age hydration products are dominated by a specific type of silicate skeleton structure, with a relatively limited variety of product types. By 28 days, the intensities of the three peaks tend to converge, with a marked increase in the intensities of the two side peaks. This evolution is primarily attributed to the continuous dissolution and structural incorporation of aluminum species from GGBFS and fly ash under the activation of carbide slag: aluminum participates in the construction of the silicate skeleton, introducing Si–O–Al vibration modes that homogenize the peak profile originally dominated by a single Si–O vibration. The incorporation of aluminum promotes the cross-linking of silicate chains, enhancing the structural ordering and compactness of the gel [36]. Furthermore, the continuous dissolution of fly ash and GGBFS supplies a sustained source of reactive silica to the system, reducing the overall Ca/Si ratio of the gel. This is mutually corroborative with the age-dependent broadening of the diffuse hump in the XRD patterns, collectively reflecting the intrinsic increase in gel polymerization and structural ordering.

#### 3.3.3. Thermal Stability and Component Content

**Figure 8:**
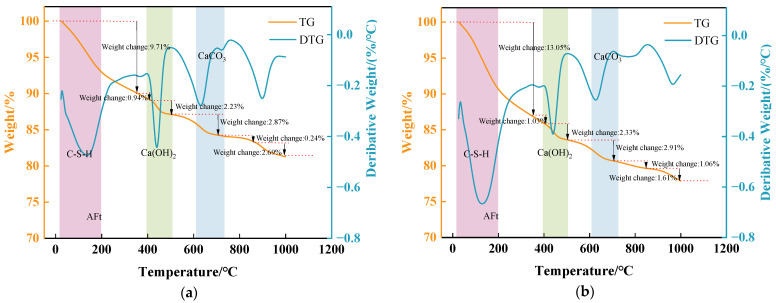
TG–DTG curves of the solid waste composite cementitious material: (**a**) 7 days; (**b**) 28 days.

Figure 8 presents the TG–DTG curves of the solid waste composite cementitious material at 7 and 28 days. The DTG curves exhibit three characteristic mass loss intervals: 80–200 °C, corresponding to the dehydration of C-(A)-S-H gel (partially overlapping with AFt); 400–500 °C, corresponding to the dehydroxylation of CH; and 620–700 °C, corresponding to the decomposition of CaCO_3_ [28,37,38,39]. With prolonged curing age, the mass loss in the 80–200 °C interval increased from 9.71% to 13.05%, suggesting that the pozzolanic reaction of GGBFS and fly ash proceeded continuously, leading to an increase in the total amount of hydration products and the chemically bound water content. The state of these products evolved from a loose and disordered form at the early stage to a dense and highly polymerized form at the later stage. The continuous improvement in macroscopic mechanical properties is precisely the direct consequence of this microstructural transformation. In the 400–500 °C interval, the mass loss due to CH dehydroxylation increased only slightly from 2.23% to 2.33%, with the increment falling within the range of instrumental error, indicating that it remained nearly constant. The mass loss due to CaCO_3_ decomposition in the 620–700 °C interval also remained essentially constant, excluding the interference of carbonation and confirming that the slight increase in CH mass loss genuinely reflects the change in the total CH content. Combined with the persistent presence of the free –OH stretching vibration band near 3440 cm^−1^ in the FTIR spectra at both curing ages, it is further confirmed that CH existed stably and continuously in the system. However, the intensity of the main CH diffraction peaks in the XRD patterns decreased significantly at 28 days. This discrepancy arises from the differing measurement principles of the two techniques: TG measures the absolute mass of CH, whereas XRD only reflects the signal of “exposed” CH that possesses an intact crystal lattice and is not covered. Therefore, the weakening of the XRD signal does not indicate a reduction in the total amount of CH, but rather results from the physical encapsulation of CH by the newly formed C-(A)-S-H gel and the lattice distortion induced by the release of Al^3+^ from FA. These complementary findings demonstrate that the generation and consumption of CH tend to reach a dynamic equilibrium at the interface, ensuring a stable alkaline activation environment at later ages.

#### 3.3.4. Microstructural Morphology and Evolution

**Figure 9:**
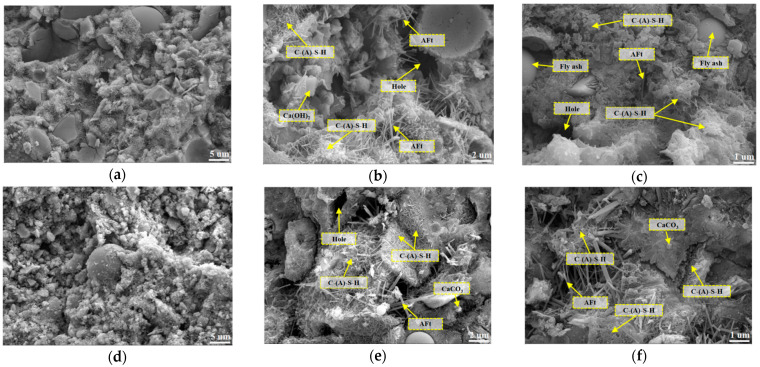
SEM micrographs of the solid waste composite cementitious material: (**a**) 7 days, 2000× magnification; (**b**) 7 days, 5000× magnification; (**c**) 7 days, 10,000× magnification; (**d**) 28 days, 2000× magnification; (**e**) 28 days, 5000× magnification; (**f**) 28 days, 10,000× magnification.

The SEM micrographs of the solid waste composite cementitious material after 7 and 28 days of curing are shown in Figure 9. At 7 days of hydration, a hydration product layer had initially formed on the particle surfaces; however, the overall structure remained relatively loose. A large number of unhydrated smooth spherical fly ash microspheres and irregularly shaped GGBFS and clinker particles were visible in the field of view. A certain amount of flocculent amorphous C-(A)-S-H gel was distributed on the particle surfaces and in the surrounding voids, consistent with the disordered gel corresponding to the diffuse hump in the XRD pattern at 7 days; however, the connection and agglomeration among the gel products were still insufficient, resulting in a loose flocculent or honeycomb-like structure, which is consistent with its low degree of polymerization. The interfacial transition zone between the particles and the gel matrix was clearly distinguishable, appearing as an annular loose band with relatively high porosity and the presence of micropores of varying sizes, which is consistent with the insufficient degree of hydration reflected by the low bound water content at 7 days in the TG–DTG analysis. In addition, sporadic needle-rod-like ettringite crystals were dispersed in the pores or on the particle surfaces. At 28 days, the microstructure underwent significant densification. The particle surfaces were extensively covered by hydration products, and the unhydrated cores were markedly reduced. The amount of flocculent C-(A)-S-H gels increased substantially, developing into a continuous and dense gel network with overlapping and intergrown coverage. This morphological feature fully corresponds to the “increasing quantity with dense structure” of the hydration products, as revealed by the broadening of the diffuse hump in XRD, the homogenization of the peak profile in FTIR, and the significant increase in bound water content in TG–DTG. These observations demonstrate that with increasing curing age, the hydration products evolved from a loose flocculent morphology to a dense gel network, with enhanced interfacial bonding and progressive pore filling. This microstructural evolution provided structural support for the improvement in macroscopic mechanical properties.

## 4. Mechanical Properties and Durability of SWCCM-SG

### 4.1. Mechanical Properties

**Figure 10:**
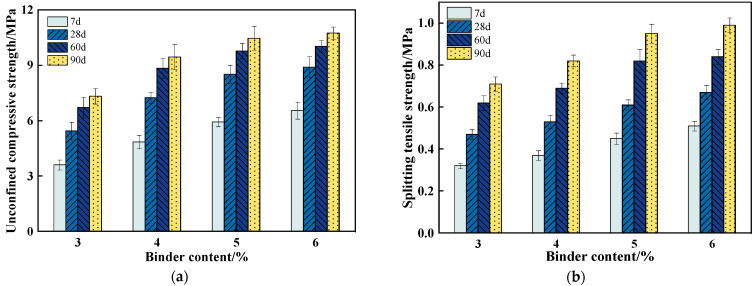
Mechanical property test results. (**a**) Unconfined compressive strength test results. (**b**) Splitting tensile strength test results.

Based on the optimal mix proportion of the solid waste composite cementitious material, the mechanical property test results of SWCCM-SG at different binder contents are presented in Figure 10.

In terms of unconfined compressive strength, as shown in Figure 10a, when the binder content was 3%, the 7-day strength of the specimens was only 3.61 MPa, failing to meet the 5.0–7.0 MPa requirement for base courses of expressways and Class I highways under extremely heavy and especially heavy traffic conditions, as specified in JTG/T F20-2015. This indicates that the active components were insufficient to support adequate early-age hydration reactions, leading to limited strength development at this binder content. When the binder content increased to 4%, the strength at each curing age improved significantly, with the 7-day strength increasing by 34.3% and the 90-day strength by 28.9% compared with those at 3% content. At a binder content of 5%, the 7-day strength reached 5.93 MPa, satisfying the specification requirements, and the 28-day strength had attained 81.4% of the 90-day strength, indicating relatively sufficient early-age hydration. When the binder content further increased to 6%, the 7-day and 28-day strengths continued to rise to 6.55 MPa and 8.91 MPa, respectively; however, the 60-day and 90-day strengths showed little difference from those at 5% content, suggesting that further increasing the binder content contributed little to the long-term strength.

As shown in Figure 10b, at a binder content of 3%, the splitting tensile strength at each curing age ranged only from 0.32 MPa to 0.71 MPa, indicating poor bonding performance of the mixture. When the binder content increased to 4%, the 7-day and 90-day splitting tensile strengths increased by 15.6% and 16.5%, respectively, compared with those at 3% content. At a binder content of 5%, the 7-day strength increased by 21.6% and the 90-day strength by 17.1% compared with those at 4% content, and the 28-day strength had reached 64.6% of the 90-day strength, indicating progressive densification of the interfacial structure of the mixture. At a binder content of 6%, the 7-day strength increased by 13.3% compared with that at 5% content, whereas the 90-day strength increased by only 0.04 MPa, representing a marginal increase of 4.2%, demonstrating limited long-term strength gain. In terms of the strength development pattern, the strength at each curing age was generally low at low binder content, whereas the strength gain at intermediate and high binder content was more pronounced at later ages, as the hydration products progressively filled the interfacial pores and the bonding performance continuously improved. In summary, the solid waste composite cementitious material at a binder content of 5% not only ensured adequate early-age strength development but also achieved long-term strength comparable to that at 6% content, offering the highest material utilization efficiency, and thus it can be recommended as the optimal binder content for this system.

### 4.2. Durability

#### 4.2.1. Drying Shrinkage Tests

**Figure 11:**
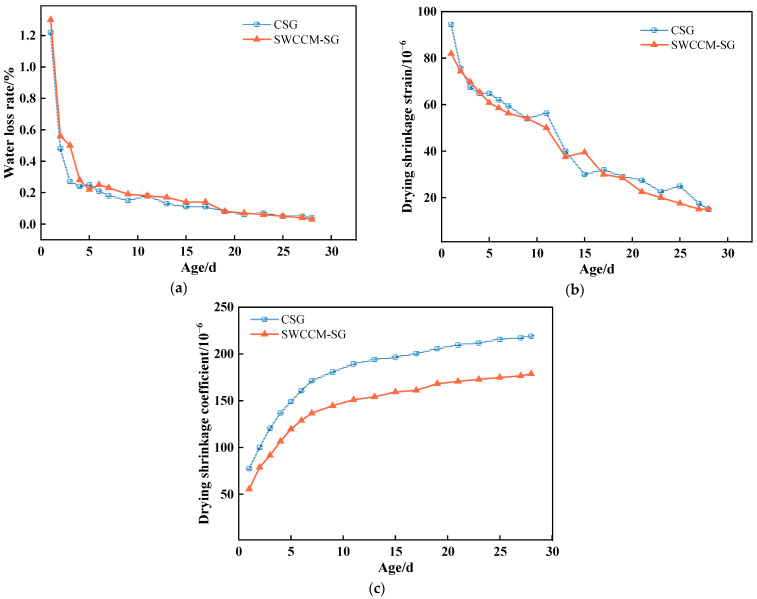
Drying shrinkage test results. (**a**) Variation in water loss rate with age. (**b**) Variation in drying shrinkage strain with age. (**c**) Variation in drying shrinkage coefficient with time.

Based on the results presented in the previous section, the binder content of the solid waste composite cementitious material was determined to be 5%. The drying shrinkage test results for the experimental group and the control group are shown in Figure 11.

As shown in Figure 11, the water loss rates of both mixtures decreased rapidly in the early stage and then tended to stabilize with increasing age. During the period of 0–4 days, free water evaporated rapidly, and the water loss rates of the two mixtures accounted for 57.2% and 61.3% of the total water loss, respectively. At 28 days of curing, the final water loss rate of SWCCM-SG was 0.03%, which was 25% lower than that of CSG. In terms of drying shrinkage strain, SWCCM-SG exhibited lower values than CSG at all curing ages. At the initial stage, the drying shrinkage strain of SWCCM-SG was 82 × 10^−6^, which was 13.2% lower than that of CSG. At 28 days, the drying shrinkage coefficients of the two mixtures decreased by 81.7% and 84.1%, respectively, compared with their initial values. With respect to the variation in drying shrinkage coefficient, both materials displayed an increasing trend with prolonged curing age. However, SWCCM-SG consistently exhibited lower values than CSG at all ages. At 28 days of curing, the drying shrinkage coefficient of SWCCM-SG was 178.90 × 10^−6^, which was 18.3% lower than that of CSG. The superior drying shrinkage performance of the solid waste composite cementitious material can be attributed to the following reasons: the C-S-H gel in the hydration products possesses relatively low shrinkage characteristics, enabling it to maintain volumetric stability during water loss. The filling effect of the hydration products refines the pore structure of the system, thereby reducing the effect of capillary tension, and the continuous hydration at later ages progressively increases the strength, providing effective restraint against shrinkage deformation. In summary, SWCCM-SG exhibited lower drying shrinkage strain and a lower drying shrinkage coefficient, indicating a smaller total shrinkage deformation and a lower shrinkage sensitivity per unit water loss. These characteristics are beneficial for reducing drying shrinkage cracking and make the material particularly suitable for engineering applications in arid and semiarid regions.

#### 4.2.2. Thermal Shrinkage Tests

To investigate the effect of replacing cement with the solid waste composite cementitious material on the thermal shrinkage behavior of stabilized gravel mixtures, the thermal shrinkage strain and thermal shrinkage coefficient of the two mixtures were measured over different temperature ranges from 40 °C to −20 °C. The results are presented in Table 9.

The thermal shrinkage test results of the two mixtures are presented in Table 8. As the temperature decreased, both the thermal shrinkage strain and the thermal shrinkage coefficient of the two mixtures exhibited a trend of first decreasing and then increasing. In the high-temperature range of 40–30 °C, the thermal shrinkage strain was the largest, with values of 77.2 × 10^−6^ for CSG and 79.8 × 10^−6^ for SWCCM-SG. In the range of 10–0 °C, the thermal shrinkage strain decreased to the minimum values of 35.9 × 10^−6^ and 35.3 × 10^−6^, representing reductions of 53.5% and 55.8%, respectively, compared with the initial stage. Upon entering the negative-temperature range, the thermal shrinkage strain increased again, reaching 46.8 × 10^−6^ and 46.5 × 10^−6^ in the range of −10 to −20 °C, respectively. In the high-temperature range, the free water content is relatively high, and the water contraction and capillary tension induced by the temperature decrease are pronounced. As the temperature continues to decrease, the free water content gradually diminishes, and the driving force for shrinkage weakens. Upon entering the negative-temperature range, the pore water freezes and undergoes volumetric expansion, leading to a renewed increase in the thermal shrinkage strain. The variation trend of the thermal shrinkage coefficient was generally consistent with that of the thermal shrinkage strain. In the range of 10–0 °C, the thermal shrinkage coefficient decreased to the minimum values of 3.59 × 10^−6^/°C and 3.53 × 10^−6^/°C, with the latter being 1.67% lower than the former. In the range of −10 to −20 °C, the values increased to 4.68 × 10^−6^/°C and 4.65 × 10^−6^/°C, respectively. The SWCCM-SG mixture exhibited slightly better thermal shrinkage performance than CSG in most temperature ranges, which can be attributed to its higher C-S-H gel content, better deformation compatibility, and a more refined and denser pore structure. In the high-temperature range of 40–30 °C, the thermal shrinkage of SWCCM-SG was slightly higher than that of CSG, which may be related to the ongoing hydration reactions and the not-yet-fully stabilized structure in this temperature range. Since the hydration products of the two mixtures are broadly similar, the difference in the thermal shrinkage coefficient was relatively small. In summary, replacing cement with the solid waste composite cementitious material does not adversely affect the thermal stability of the base course material and may even result in a slight improvement.

#### 4.2.3. Freeze–Thaw Cycle Test

**Figure 12:**
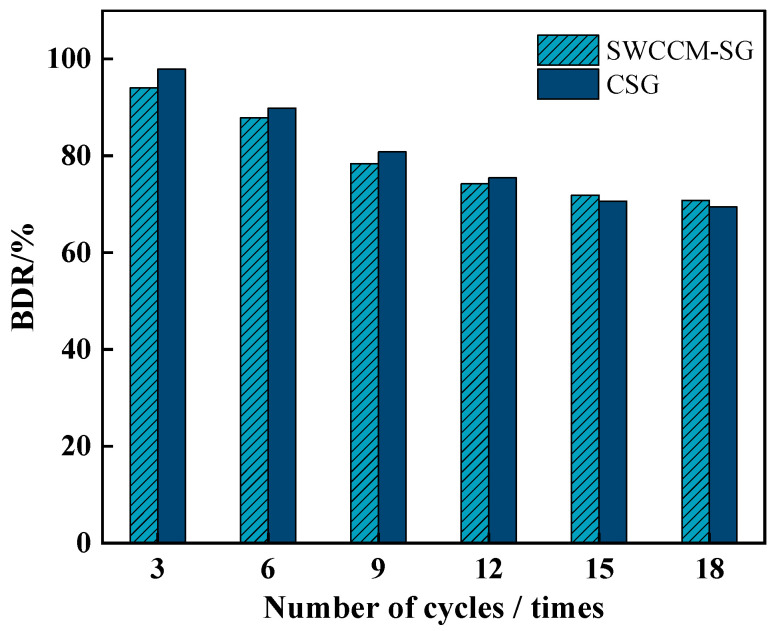
Strength retention ratio of the two mixtures after freeze–thaw cycles.

The compressive strength of the SWCCM-SG and CSG mixtures after different numbers of freeze–thaw cycles is shown in Figure 12.

With the increase in the number of freeze–thaw cycles, the BDR (freezing durability ratio) values of both mixtures exhibited a continuous decreasing trend, but the rates of change varied at different stages. In the early stage of freeze–thaw cycling, the BDR value of CSG was 97.96%, representing a strength loss of only 2.04%. The BDR value of SWCCM-SG was 94.25%, corresponding to a strength loss of 5.75%, which was 3.71% higher than that of CSG. As the number of freeze–thaw cycles increased to six, the BDR value of CSG decreased to 89.81%, representing a reduction of 8.3% compared with that at three cycles. The BDR value of SWCCM-SG decreased to 87.95%, representing a reduction of 6.7% compared with that at three cycles. When the number of freeze–thaw cycles reached nine, the BDR value of SWCCM-SG exceeded that of CSG for the first time. This turning point indicates that after a certain number of freeze–thaw cycles, the frost resistance advantage of the solid waste system began to manifest. As the number of freeze–thaw cycles further increased to 12, 15, and 18, the BDR values of both mixtures continued to decline. However, SWCCM-SG consistently remained at a higher level.

**Figure 13:**
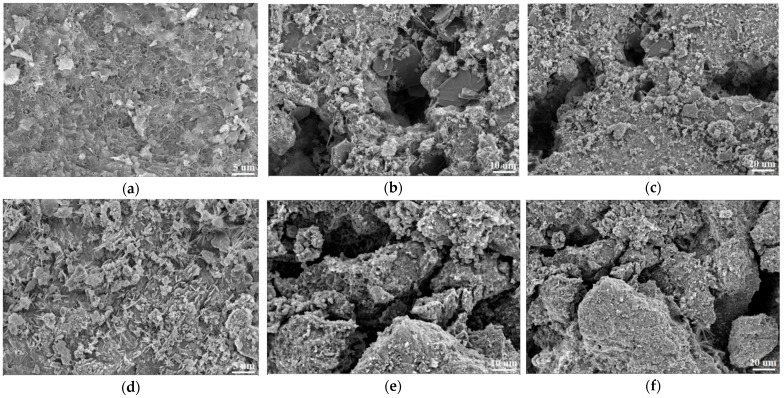
Comparison of microstructural morphology. (**a**) SWCCM-SG before freeze–thaw cycles, 2000× magnification; (**b**) SWCCM-SG after freeze–thaw cycles, 1000× magnification; (**c**) SWCCM-SG after freeze–thaw cycles, 500× magnification; (**d**) CSG before freeze–thaw cycles, 2000× magnification; (**e**) CSG after freeze–thaw cycles, 1000× magnification; (**f**) CSG after freeze–thaw cycles, 500× magnification.

The microstructures of the two mixtures before and after freeze–thaw cycling are shown in Figure 13. Before freeze–thaw cycling, both mixtures exhibited a dense structure. In SWCCM-SG, the cementitious matrix was tightly bonded with the aggregates, the interfacial transition zone was continuous and intact, and the flocculent hydration products were uniformly distributed. CSG also exhibited a continuous structure without apparent initial defects. After 18 freeze–thaw cycles, significant differences between the two mixtures were observed. CSG displayed penetrating, wide cracks, with the aggregate–paste interface clearly exposed and the structure becoming loose and fragmented. High-magnification observations revealed debris particles accumulated along the crack edges, fractured needle-rod-like ettringite crystals, and blurred edges of plate-like calcium hydroxide crystals due to dissolution, indicating that highly crystalline hydration products underwent fracturing and dissolution under frost heave stress. In contrast, the SWCCM-SG mixture maintained excellent microstructural integrity, with a dense matrix, aggregates tightly encapsulated by the cementitious material, and no penetrating cracks. High-magnification observations elucidated the underlying durability mechanism: the surface was extensively covered with an interwoven network of flocculent C-(A)-S-H gel, and fine cracks were sparse and were filled and blunted by the gel. This microstructure, rich in low-Ca/Si C-S-H gel, optimizes the pore characteristics through dense filling and effectively buffers the frost heave stress through the toughness and adhesion of the gel, thereby inhibiting microcrack propagation. In summary, the solid waste composite cementitious material, with its low-calcium hydration product composition and dense microstructural network, demonstrates a significant advantage over conventional cement in resisting freeze–thaw damage.

**Figure 14:**
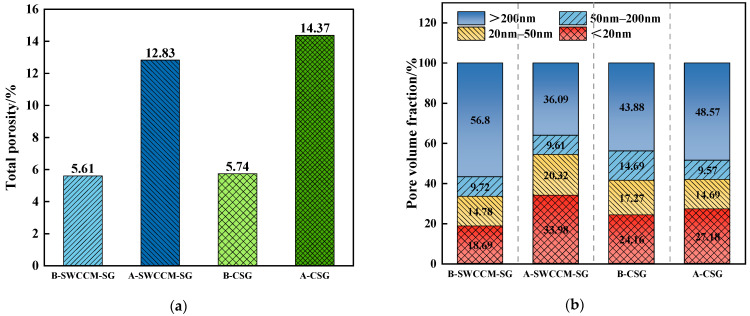
Comparison of microscopic pore characteristics. (**a**) Total porosity of the two mixtures before and after 18 freeze–thaw cycles. (**b**) Pore volume ratio of the two mixtures before and after 18 freeze–thaw cycles.

The changes in porosity and pore size distribution of the two mixtures before and after freeze–thaw cycling are shown in Figure 14 (where “B” denotes the state before freeze–thaw cycling, e.g., B-SWCCM-SG and B-CSG, and “A” denotes the state after freeze–thaw cycling). Before freeze–thaw cycling, the total porosities of SWCCM-SG and CSG were 5.61% and 5.74%, respectively, which were relatively close in value. After 18 freeze–thaw cycles, the total porosity of both mixtures increased, but the magnitudes of increase differed significantly: that of SWCCM-SG increased to 12.83%, while that of CSG increased to 14.37%, with the latter being 12% higher than the former, indicating more severe internal structural damage in CSG.

In terms of pore size distribution, prior to freeze–thaw cycling, the SWCCM-SG mixture was dominated by macropores, accounting for 56.80%, whereas the macropore proportion in the CSG mixture was 43.88%. After freeze–thaw cycling, the pore size distributions of the two mixtures exhibited distinctly different trends. SWCCM-SG displayed a micro-refinement characteristic of “reduced macropores and increased micropores.” The proportion of macropores decreased from 56.80% to 36.09%, while the proportion of ultrafine micropores increased from 18.69% to 33.98%, indicating that macropores were effectively filled during the freeze–thaw process and that the pore structure tended to become more refined. In contrast, CSG exhibited a deterioration characteristic of “increased macropores and reduced micropores.” The proportion of macropores increased from 43.88% to 48.57%, while the proportions of micropores and mesopores decreased by 14.94% and 34.85%, respectively, suggesting that some micropores and mesopores became interconnected and evolved into larger pores.

The above differences can be attributed to the distinct hydration product compositions. In the CSG mixture, the calcium hydroxide crystals are relatively brittle and susceptible to fracturing under frost heave stress, and the dissolution and recrystallization of ettringite may also exacerbate the structural damage. In contrast, the SWCCM-SG mixture is dominated by C-S-H gel, which possesses good toughness and filling capacity, enabling it to effectively buffer frost heave stress and optimize the pore structure.

#### 4.2.4. Wet–Dry Cycle Test

**Figure 15:**
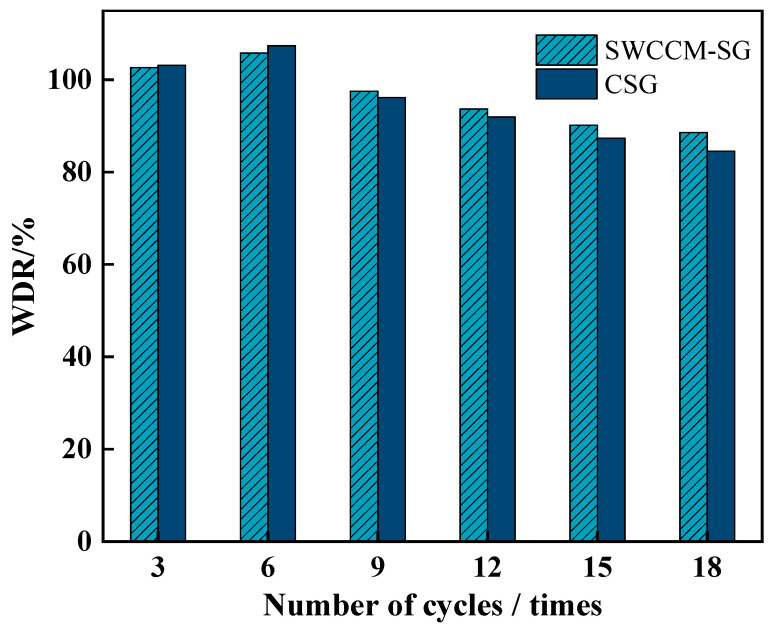
Strength retention ratio of the two mixtures after wet–dry cycles.

The variation in wetting–drying resistance (WDR) values of the two mixtures with the number of wet–dry cycles is shown in Figure 15. Both mixtures exhibited a trend of first increasing and then decreasing. In the early stage of the test (three to six cycles), the BDR values of both mixtures exceeded 100%: that of CSG increased from 103.09% to 107.34%, while that of SWCCM-SG increased from 102.63% to 105.79%. This phenomenon is attributed to the repeated migration of moisture during the wet–dry alternation, which promoted the continuous hydration of the cementitious materials, generating more C-S-H gel to fill the pores and thereby enhancing the structural compactness. After 18 cycles, the WDR value of CSG decreased by 21.3% compared with its peak value, with a cumulative strength loss of 15.53%; the WDR value of SWCCM-SG decreased by 16.2% compared with its peak value, with a cumulative strength loss of 11.26%. The more rapid strength deterioration of CSG in the later stage is primarily due to the susceptibility of calcium hydroxide in its hydration products to dissolution and leaching under alternating wet–dry conditions, leading to structural loosening. In contrast, the SWCCM-SG mixture is dominated by C-S-H gel, with a lower calcium hydroxide content. The gel phase possesses good water stability, and the unreacted particles continue to exert their pozzolanic reaction potential during the wet–dry cycles, providing dynamic compensation for the strength loss. As a result, SWCCM-SG demonstrates superior resistance to wet–dry damage.

**Figure 16:**
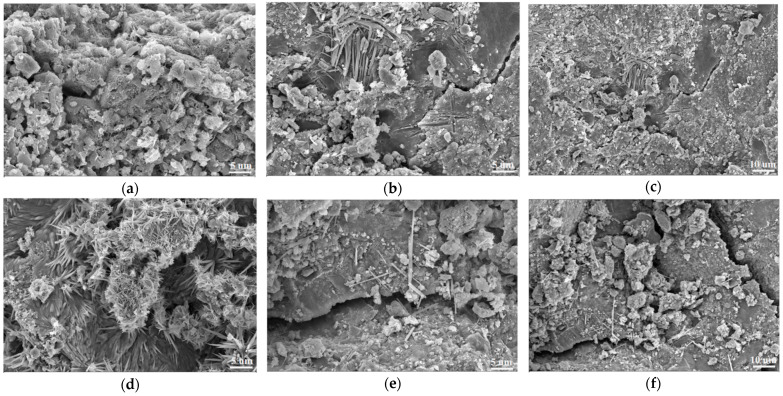
Comparison of microstructural morphology after wet–dry cycles. (**a**) SWCCM-SG before wet–dry cycles, 2000× magnification; (**b**) SWCCM-SG after wet–dry cycles, 2000× magnification; (**c**) SWCCM-SG after wet–dry cycles, 1000× magnification; (**d**) CSG before wet–dry cycles, 2000× magnification; (**e**) CSG after wet–dry cycles, 2000× magnification; (**f**) CSG after wet–dry cycles, 1000× magnification.

The microstructures of the two mixtures before and after the wet–dry cycle are shown in Figure 16. Before the wet–dry cycle, both mixtures exhibited a relatively dense structure. In SWCCM-SG, the cementitious matrix was tightly bonded with the aggregates. No apparent cracks were observed in the interfacial transition zone, and the hydration products covered the particle surfaces and filled the pores in a flocculent form. CSG also displayed a continuous and dense microstructure. After 18 wet–dry cycles, significant differences between the two mixtures were observed. At 2000× magnification, distinct microcracks appeared in the CSG matrix, exhibiting an irregular distribution with partial connectivity. Debonding was observed in the interfacial transition zone, and the overall structure tended to become loose. In contrast, although a small number of microcracks were present in the SWCCM-SG mixture, the crack dimensions were relatively small, and no penetrating damage was formed. The gel matrix remained relatively intact, the aggregates were tightly encapsulated, and the interfacial bonding was favorable. At 1000× magnification, evident dissolution traces were observed on the surface of the hydration products in CSG, with increased porosity in some regions and pronounced structural loosening. In the SWCCM-SG mixture, the gel matrix remained dense, with the flocculent gel network interwoven into a continuous structure without apparent signs of dissolution. The overall microstructural integrity was markedly superior to that of the cement-based material.

**Figure 17:**
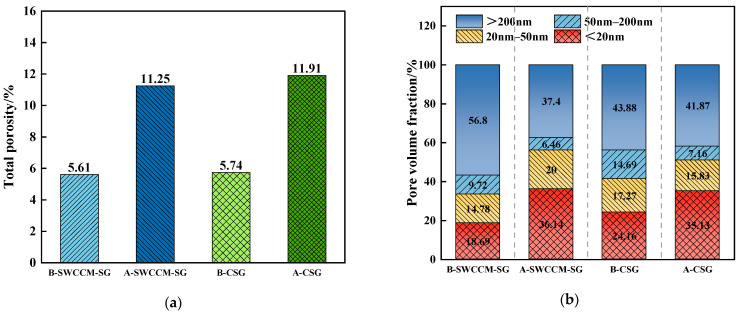
Comparison of pore characteristics after wet–dry cycles. (**a**) Total porosity of the two mixtures before and after 18 wet–dry cycles. (**b**) Pore volume ratio of the two mixtures before and after 18 wet–dry cycles.

The changes in porosity and pore size distribution of the two mixtures before and after wet–dry cycling are shown in Figure 17 (where “B” denotes the state before wet–dry cycle, e.g., B-SWCCM-SG and B-CSG, and “A” denotes the state after wet–dry cycle, e.g., A-SWCCM-SG and A-CSG). Before the wet–dry cycle, the total porosities of SWCCM-SG and CSG were 5.61% and 5.74%, respectively, which were essentially comparable at the initial stage. After 18 wet–dry cycles, the total porosity of SWCCM-SG increased to 11.25%, while that of CSG increased to 11.91%, with the latter being 5.54% higher than the former, indicating more severe internal structural damage in the CSG mixture.

In terms of pore size distribution, the two mixtures exhibited distinctly different evolution paths. SWCCM-SG displayed a trend in which the proportion of macropores decreased by 34.15%, the proportion of ultrafine micropores increased from 18.69% to 36.14%, and the proportion of micropores increased by 35.32%, reflecting a micro-refinement characteristic of macropores transforming into smaller pores. In contrast, CSG exhibited a trend in which the proportion of macropores decreased by only 4.58%, the proportion of ultrafine micropores increased from 24.16% to 35.13%, the proportion of mesopores decreased from 14.69% to 7.16%, and the proportion of micropores decreased by 8.34%, reflecting pore connectivity and coarsening characteristics.

The pore structure optimization of “macropore filling and micropore increase” achieved by SWCCM-SG after the wet–dry cycle is the microstructural essence underlying its significantly lower cumulative strength loss compared with CSG. As harmful pores, the decreased proportion of macropores implies the elimination of stress concentration sources, while the increased proportion of ultrafine micropores serves as an indicator of C-S-H gel formation and system densification. The difference in pore structure between the two mixtures stems from their distinct hydration product compositions. In CSG, the calcium hydroxide crystals are prone to dissolution and leaching during the wetting stage, compromising the interfacial bonding. Furthermore, the repeated changes in capillary pressure induce microcrack propagation and pore coarsening. In SWCCM-SG, C-S-H gel dominates the hydration products, with an extremely low calcium hydroxide content, rendering the dissolution effect negligible. Instead, the wet–dry alternation activates the pozzolanic reaction of unreacted particles, and the continuously generated gel preferentially fills the macropores, thereby optimizing the pore structure toward micro-refinement.

## 5. Conclusions

In this study, a solid waste composite cementitious material was prepared using three types of industrial solid waste—GGBFS, FA, and CS—as the main raw materials. The mix proportions were optimized through single-factor experiments and response surface methodology. The hydration mechanism, microstructure, and mechanical properties and durability of the resulting stabilized gravel base course were systematically investigated. The main conclusions are as follows.

(1) The single-factor experiments indicated that the 28-day compressive strength reached a peak value of 38.58 MPa at an FA content of 30%, and peaked at a GGBFS content of 40%, which was 17.0% higher than that at 60% content. For CS, the 28-day compressive strength reached a peak value of 37.83 MPa at a content of 4%. The influence of each factor on strength initially increased, then decreased. Through single-factor experiments combined with Box–Behnken response surface optimization, the optimal mix proportion of the solid waste composite cementitious material was determined to be FA 21.0%, GGBFS 36.3%, and CS 5.7%, corresponding to a 28-day compressive strength of 42.93 MPa, a 28-day flexural strength of 13.02 MPa, and a fluidity of 184.1 mm. The errors between the model predictions and the experimental results were less than 5%, confirming the reliability of the optimization results.

(2) During the curing period from 7 to 28 days, the solid waste composite cementitious material exhibited a broadening of the diffuse hump in the XRD pattern of the C-(A)-S-H gel, a homogenization of the FTIR peak profiles, an increase in the mass loss of water from 9.71% to 13.05%, and a microstructural transformation in SEM morphology from a loose flocculent structure to a continuous dense network. These observations collectively indicate a transition of the gel structure from a low-polymerization disordered state to a high-polymerization ordered state, confirming the continuous increase in the total amount of hydration products. CH maintained the alkaline environment through a dynamic equilibrium between its generation and consumption, driving the continuous progression of hydration. The quantitative growth and qualitative evolution of the microstructure jointly contributed to the improvement ine macroscopic mechanical properties.

(3) The mechanical properties of SWCCM-SG improved with increasing binder content. As the binder content increased from 3% to 5%, the 7-day unconfined compressive strength increased from 3.61 MPa to 5.93 MPa, satisfying the strength requirements for base courses of expressways and Class I highways. Moreover, the 28-day strength had attained 81.4% of the 90-day strength, indicating rapid early-age strength development. When the binder content further increased to 6%, the 28-day strength increased to 8.91 MPa; however, the 90-day strength was essentially comparable to that at 5% content, indicating limited long-term strength gain. The splitting tensile strength exhibited a similar trend. Considering both material utilization efficiency and performance, 5% was determined to be the optimal recommended binder content for the solid waste composite cementitious material.

(4) SWCCM-SG comprehensively outperformed CSG in terms of durability. In terms of drying shrinkage performance, the 28-day water loss rate of SWCCM-SG was 25% lower and the drying shrinkage coefficient was 18.3% lower than those of CSG. The thermal shrinkage performance of SWCCM-SG was essentially comparable to that of CSG, indicating that replacing cement with the solid waste composite cementitious material does not adversely affect the thermal shrinkage behavior. In terms of frost resistance, the strength retention ratio of SWCCM-SG after 18 freeze–thaw cycles was 2.4% higher than that of CSG and the increase in total porosity was 12% lower. The pore structure exhibited a micro-refinement trend of “reduced macropores and increased micropores,” which is attributed to the toughness and stress-buffering capacity of the C-S-H gel and the continuous filling effect of the pozzolanic reaction. In terms of resistance to wet–dry damage, the cumulative strength loss of SWCCM-SG after 18 cycles was 11.26%, which was superior to the 15.53% of CSG, and the increase in total porosity was 5.54% lower. The pore structure displayed micro-refinement characteristics, and the wet–dry alternation activated the pozzolanic reaction to generate C-S-H gel that filled the macropores, providing dynamic compensation for the damage.

## Figures and Tables

**Table 1:** Main chemical composition of the test cement

Constituent/%	SiO_2_	Al_2_O_3_	Fe_2_O_3_	CaO	MgO	SO_3_	K_2_O	LOI
Content/(wt%)	22.16	4.55	3.04	61.37	2.43	2.74	0.69	3.02

**Table 2:** Physical and mechanical properties of cement

Fineness/%	Standard Consistency/%	Setting Time/min		Compressive Strength/MPa		Flexural Strength/MPa	
		Initial	Final	3 Days	28 Days	3 Days	28 Days
20	27.2	212	334	20.7	45.8	4.9	10.3

**Table 3:** Chemical composition of solid waste materials

Type	CaO	SiO_2_	Al_2_O_3_	Fe_2_O_3_	SO_3_	K_2_O	Na_2_O	MgO
GGBFS	42.87	31.38	11.67	0.65	0.18	0.53	1.27	6.41
FA	10.78	49.74	23.82	4.03	1.47	1.12	4.91	4.13
CS	79.52	4.86	3.14	0.87	1.52	0.08	-	0.83

**Table 4:** Properties of the aggregate used in this study

Property	Unit	Particle Size Range/mm				Technical Requirement
		20–30	15–20	10–15	5–10	
Crushing value	%	-	-	15.8	-	≤26
Los Angeles abrasion loss	%	6.5				≤28
Apparent relative density	g/cm^3^	2.893	2.882	2.915	2.887	≥2.6
Bulk relative density	g/cm^3^	2.653	2.63	2.68	2.634	-
Water absorption	%	0.56	0.61	0.54	0.86	≤2
Flat and elongated particle content	%	5.8	6.7	7.6	7.3	≤15

**Table 5:** Mix proportions for single-factor experiments

Code	FA/%	GGBFS/%	Cement/%	CS/%
F1	10	40	50	4
F2	20	40	40	4
F3	30	40	30	4
F4	40	40	20	4
F5	50	40	10	4
S1	30	20	50	4
S2	30	30	40	4
S3	30	40	30	4
S4	30	50	20	4
S5	30	60	10	4
C1	30	40	30	1
C2	30	40	30	2
C3	30	40	30	4
C4	30	40	30	6
C5	30	40	30	8

**Table 6:** Factors and levels for response surface design

Factor	Level		
	−1	0	1
FA/%	20	30	40
GGBFS/%	30	40	50
CS/%	2	4	6

**Table 7:** Summary of compaction test results

Binder Content/%	3	4	5	6
Maximum dry density/(g/cm^3^)	2.3292	2.3443	2.3595	2.3746
Optimum moisture content/%	5.45	5.67	5.86	6.02

**Table 8:** ANOVA results for the response surface models

	Y_1_		Y_2_		Y_3_	
Source	F-Value	*p*-Value	F-Value	*p*-Value	F-Value	*p*-Value
Model	78.96	<0.0001	116.03	<0.0001	97.09	<0.0001
*X_1_*	292.68	<0.0001	526.53	<0.0001	47.07	0.0002
*X_2_*	54.34	0.0002	252.52	<0.0001	15.56	0.0056
*X_3_*	71.09	<0.0001	54.27	0.0002	467.86	<0.0001
*X_1_ X_2_*	0.1153	0.7442	6.18	0.0418	60.89	0.0001
*X_1_ X_3_*	3.56	0.1011	4.32	0.0764	143.68	<0.0001
*X_2_ X_3_*	29.51	0.0010	15.98	0.0052	9.71	0.0170
*X_1_^2^*	83.71	<0.0001	33.34	0.0007	103.87	<0.0001
*X_2_^2^*	88.04	<0.0001	119.00	<0.0001	6.26	0.0409
*X_3_^2^*	95.30	<0.0001	35.00	0.0006	25.29	0.0015
Lack of fit	0.2619	0.8501	0.4072	0.7568	1.90	0.2706
*Adjusted R^2^*	0.9777		0.9848		0.9818	
*Predicted R^2^*	0.9616		0.9671		0.9201	

**Table 9:** Thermal shrinkage test results

Mixture Type	Property	Temperature Range/°C						Average Thermal Shrinkage Coefficient
		40–30	30–20	20–10	10–0	0 to −10	−10 to −20	
CSG	Thermal shrinkage strain/(10^−6^)	77.2	52.3	41.1	35.9	43.6	46.8	5.11
	Thermal shrinkage coefficient/(10^−6^/°C)	7.72	5.23	4.11	3.59	4.36	4.68	
SWCCM-SG	Thermal shrinkage strain/(10^−6^)	79.8	51.7	40.4	35.3	43.1	46.5	5.09
	Thermal shrinkage coefficient/(10^−6^/°C)	7.98	5.17	4.04	3.53	4.31	4.65	

## Data Availability

The original contributions presented in this study are included in the article. Further inquiries can be directed to the corresponding author.
